# Concise and Stereodivergent Synthesis of Carbasugars Reveals Unexpected Structure-Activity Relationship (SAR) of SGLT2 Inhibition

**DOI:** 10.1038/s41598-017-05895-9

**Published:** 2017-07-17

**Authors:** Wai-Lung Ng, Ho-Chuen Li, Kit-Man Lau, Anthony K. N. Chan, Clara Bik-San Lau, Tony K. M. Shing

**Affiliations:** 1 Department of Chemistry and Centre of Novel Functional Molecules, The Chinese University of Hong Kong, Shatin, New Territories, Hong Kong SAR, China; 2 Institute of Chinese Medicine and State Key Laboratory of Phytochemistry and Plant Resources in West China, The Chinese University of Hong Kong, Shatin, New Territories, Hong Kong SAR, China; 3 Department of Chemistry, Chemistry Research Laboratory, University of Oxford, Mansfield Road, Oxford, OX1 3TA UK; 4 Department of Medicine, Harvard Medical School, Boston, Massachusetts, 02115 USA

## Abstract

Carbasugar sodium-glucose cotransporter 2 (SGLT2) inhibitors are highly promising drug candidates for the treatment of Type 2 diabetes mellitus (T2DM). However, the clinical usage of carbasugar SGLT2 inhibitors has been underexplored, due to the lengthy synthetic routes and the lack of structure-activity relationship (SAR) studies of these compounds. Herein, we report a concise and stereodivergent synthetic route towards some novel carbasugar SGLT2 inhibitors, featuring an underexploited, regioselective, and stereospecific palladium-catalyzed allyl-aryl coupling reaction. This synthetic strategy, together with computational modeling, revealed the unexpected SAR of these carbasugar SGLT2 inhibitors, and enabled the discovery of a highly selective and potent SGLT2 inhibitor.

## Introduction

Carbasugars are carbocyclic analogues of carbohydrates, in which the endocyclic oxygen atom of the sugar core was substituted with a methylene unit^1^. Carbasugars have attracted extensive synthetic, conformational, and biological studies since the 1960s^1^. Their inhibitory activities towards carbohydrate-processing enzymes, such as α-amylase^2^, trehalase^3, 4^, α-glucosidase^5, 6^, and glycosyltransferase^7, 8^, have been well explored.

**Figure 1:**
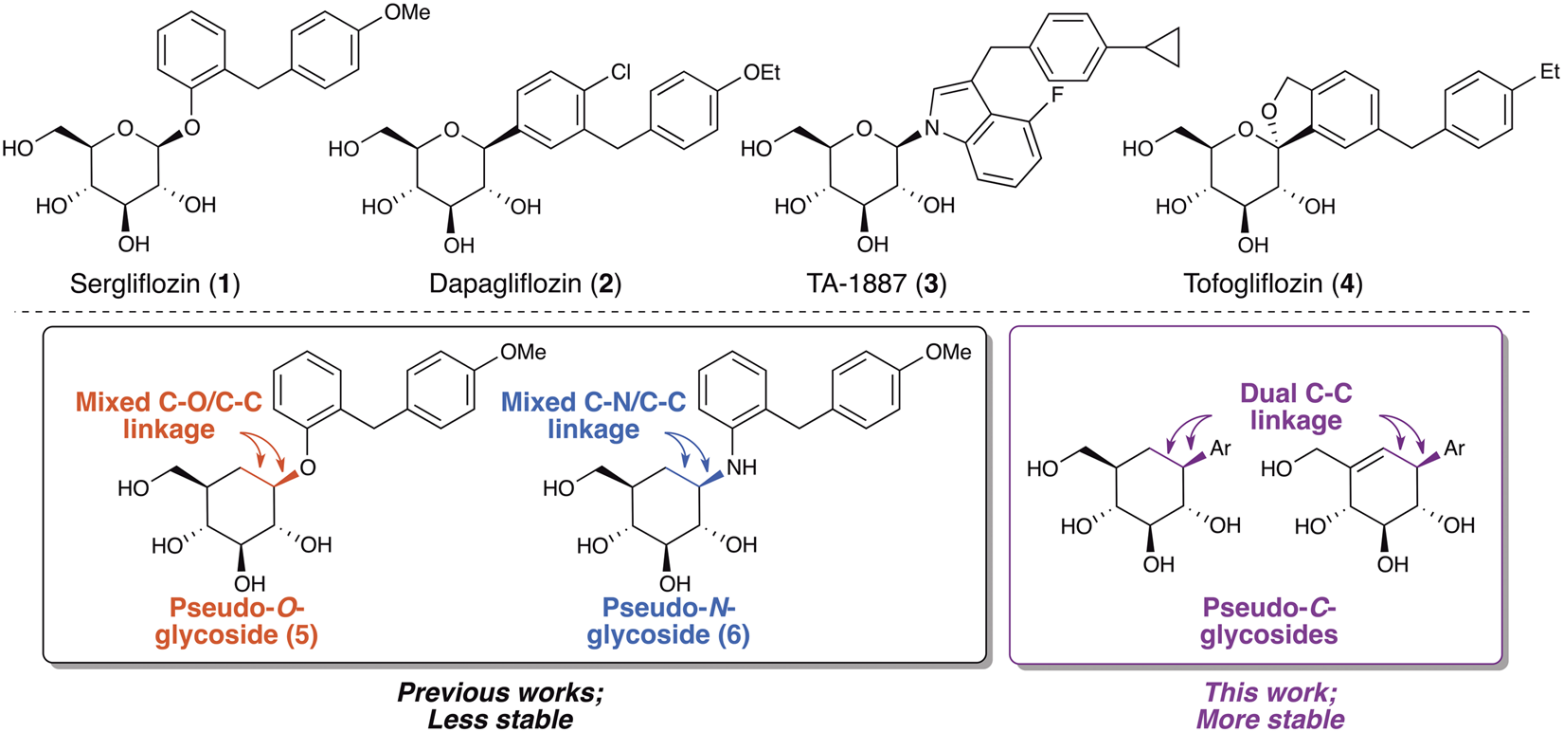
Current SGLT2 inhibitors & carbasugar SGLT2 inhibitors.

Recently, inhibition of sodium-glucose cotransporter 2 (SGLT2), a transporter protein expressed in the kidneys, has emerged as a promising treatment of diabetes *via* an insulin-independent mechanism^9–12^. Most current SGLT2 inhibitors are carbohydrate-based small molecules such as *O*-, *C*-, and mixed-glycosides (Compounds **1**–**4**, Fig. 1)^13–24^ and many recent studies have focused intensely on modifications of the aglycone unit of *C*-glycoside analogues^9–11^. Departing from this trend, we have pioneered the design and syntheses of carbasugar SGLT2 inhibitors, in which we modify the sugar core rather than the aglycone^25, 26^. We found that some of the carbasugar analogues of sergliflozin (**1**), such as pseudo-*O*-glycoside **5** and pseudo-*N*-glycoside **6**, exert a high inhibitory activity/selectivity towards SGLT2^25, 26^, and also exhibit a prolonged blood glucose lowering effect^25^.

After the initial success of **5** & **6**, we set out to further expand the synthetic toolkit towards carbasugars, and also to enhance the drug-like properties of carbasugar SGLT2 inhibitors. We postulated that aryl pseudo-*C*-glycosides, in which a C(sp^2^)–C(sp^3^) bond replace a C–O or C–N bond as in **5** & **6**, are superior carbasugar SGLT2 inhibitors as they possess higher stability due to the presence of a “dual C–C linkage” (Fig. 1, lower)^27^. We report herein a concise and stereodivergent synthetic route towards these challenging synthetic targets, and also the unexpected SAR of carbasugar SGLT2 inhibition.

## Results and Discussions

### Allyl-aryl cross-coupling route towards carbasugars

**Figure 2:**
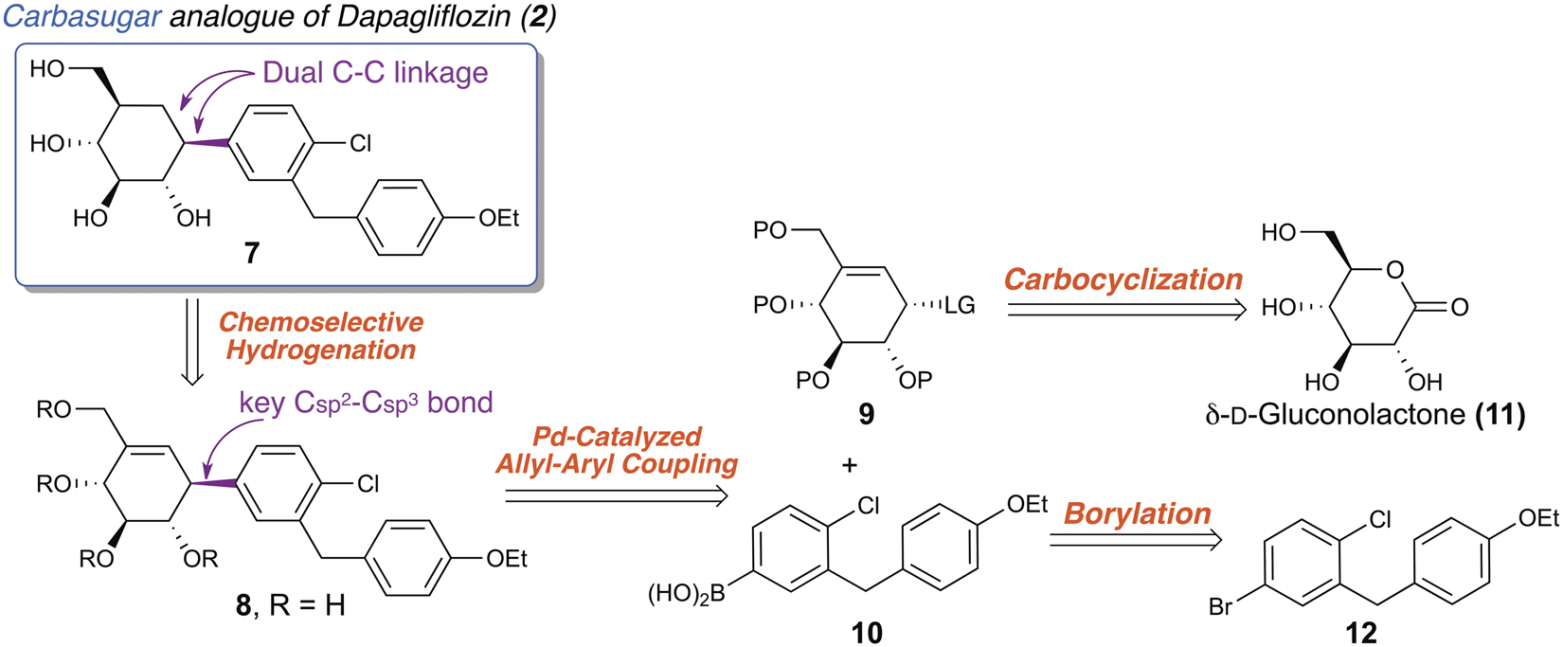
Retrosynthetic analysis of aryl pseudo-*C*-glycoside **7**.

Retrosynthetic analysis of our synthetic target **7** shows that it is readily accessible from simple starting materials such as D-gluconolactone (**11**) and arene **12** (Fig. 2). However, a major synthetic challenge is the stereoselective construction of the key C(sp^2^)–C(sp^3^) bond between the carbasugar core and aglycone. We therefore utilized an underexploited, stereospecific palladium-catalyzed allyl-aryl coupling reaction to address this challenge.

**Figure 3:**
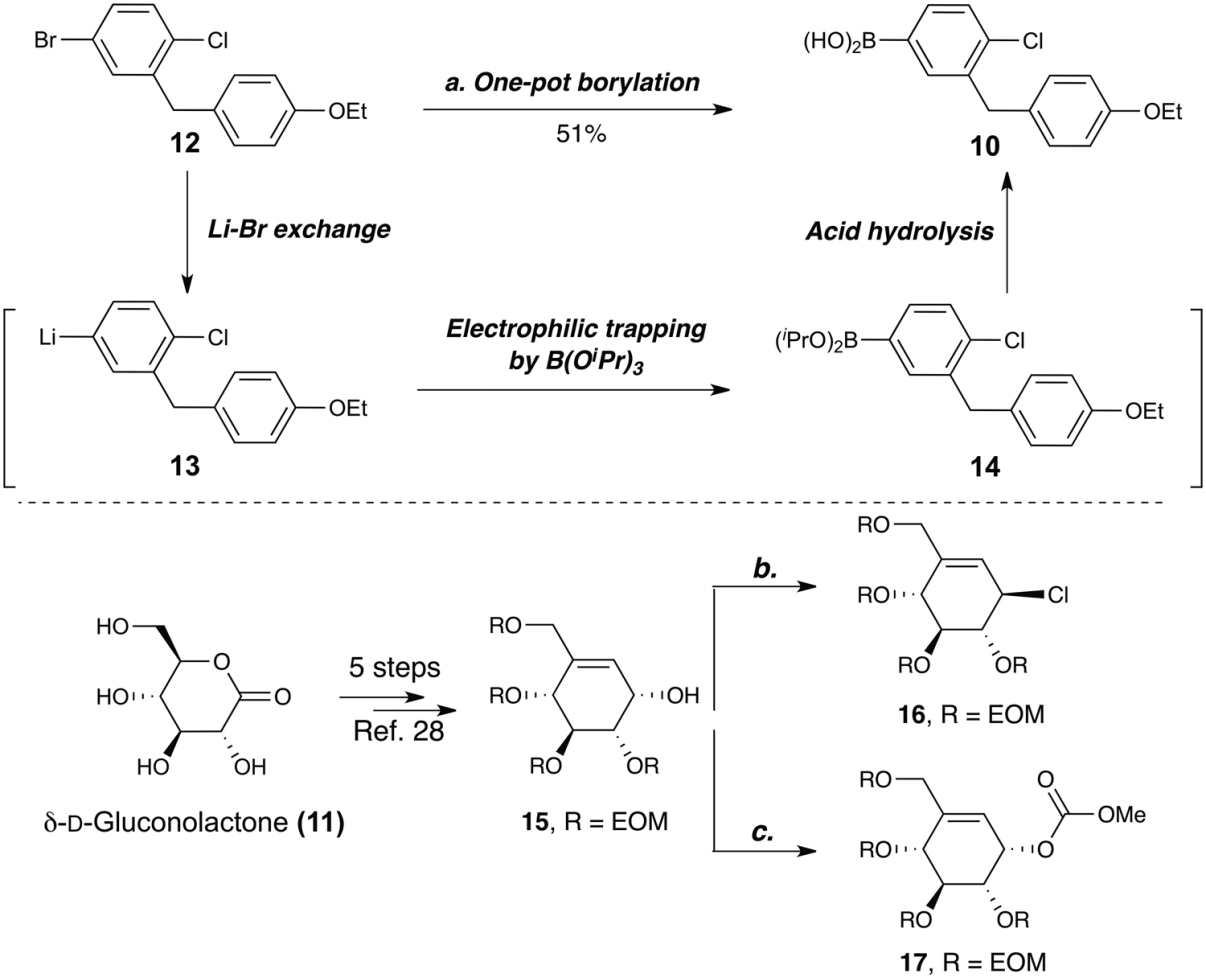
Syntheses of aryl boronic acid **10** and allylic electrophiles **16** & **17** as precursors for the subsequent coupling reactions. (**a**) 1) ^*n*^BuLi, THF:Toluene = 1:4, −78 °C; 2) B(O^i^Pr)_3_, −78 °C to −20 °C; 3) HCl_(aq)_, −20 °C to rt; (**b**) MsCl, Et_3_N, 3 A MS^*n*^, Bu_4_NCl, CH_2_Cl_2_, 81%; (**c**) Methyl chloroformate, pyridine, 0 °C to rt, 74% (92% BORSM).

We first set out to synthesize aryl boronic acid **10** as a coupling precursor (Fig. 3, upper). The commercially available bromochlorobenzene **12** was converted into aryl boronic acid **10** by a simple one-pot, three-step borylation sequence. A lithium-bromide exchange yielded the lithiated intermediate **13**. Electrophilic trapping of **13** by triisopropyl borate afforded the boronic ester **14**, which was hydrolyzed immediately upon acidic quenching to give aryl boronic acid **10**.

Next, we prepared the allylic electrophiles **16** and **17** for the subsequent allyl-aryl coupling reaction (Fig. 3, lower). Both of them were obtained from a common intermediate, allylic alcohol **15**, which is readily accessible from the commercially available, inexpensive sugar **11** ^28^.

**Figure 4:**
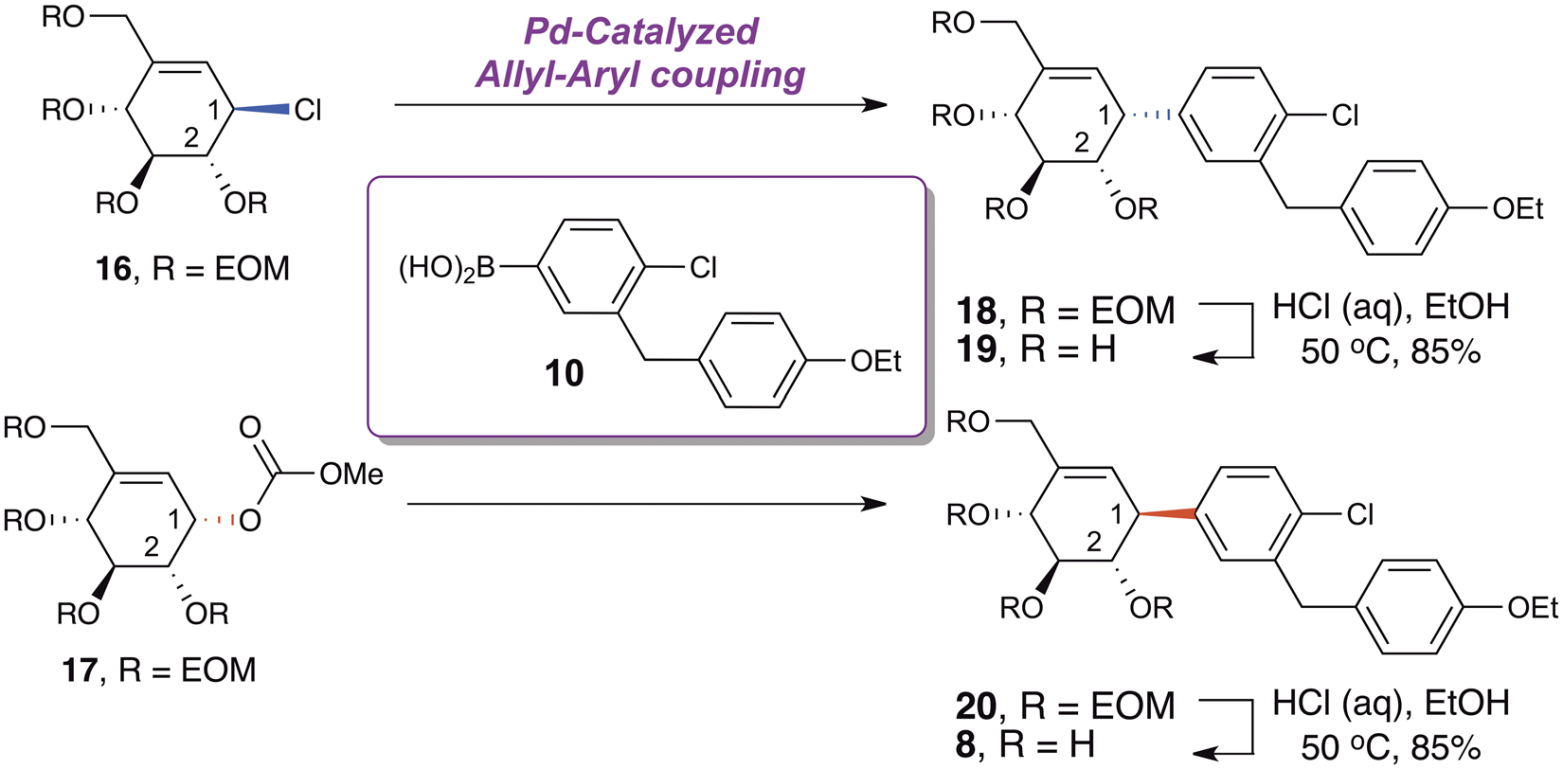
Syntheses of cyclohexene analogues of dapagliflozin *via* allyl-aryl coupling.

With the coupling precursors in hand, we attempted the key Pd-catalyzed allyl-aryl coupling reaction, which has been underexploited in the literature (Fig. 4 and Table 1)^29^. This seemingly simple transformation, however, was not trivial, and required extensive optimization. The allylic electrophiles **16** and **17** were prone to β-elimination which led to the formation of diene side products. A literature search revealed that allyl-aryl coupling reactions in cyclic systems are rare and challenging^30–32^, while those in acyclic systems often necessitate elevated reaction temperatures^33–35^. Thus, a mild reaction condition for cyclic substrates is highly sought-after. Upon extensive reaction optimization, we successfully identified that the combination of Pd(dba)_2_/K_2_CO_3_/1,4-dioxane gave the best reaction outcome for both allylic electrophiles **16** & **17** (Table 1, entries 5 & 10). Notably, the proper choice of solvent, the absence of phosphine ligand, and the mild reaction temperature are critical to a high coupling yield. Remarkably, this phosphine-free Pd-catalyzed allyl-aryl coupling reaction is stereospecific and regioselective, with arylated C-1-α-cyclohexene **18** and C-1-β-cyclohexene **20** obtained as single products.

**Table 1:** Selected reaction conditions for Pd-catalyzed allyl-aryl coupling^*a*^.

Entry	Reaction conditions	Results
1	**16**, Pd(dba)_2_, K_2_CO_3_, CHCl_3_	**18**: 38%
2	**16**, Pd(dba)_2_, K_3_PO_4_, CHCl_3_	**18**: 69%
3	**16**, Pd(dba)_2_, KF, CHCl_3_	**18**: 62%
4	**16**, Pd(dba)_2_, K_3_PO_4_, 1,4-dioxane	**18**: 74%
**5**	**16, Pd(dba)**_**2**_ **, K**_**2**_ **CO**_**3**_ **, 1,4-dioxane**	**18: 88%**
6	**17**, Pd(dba)_2_, K_2_CO_3_, CHCl_3_	**20**: 38%
7	**17**, Pd(dba) _2_, Na_2_CO_3_, 1,4-dioxane	**20**: 70%
8	**17**, Pd(dba) _2_, K_2_CO_3_, CH_3_CN	**20**: 22%
9	**17**, Pd(dba) _2_, K_2_CO_3_, 1,4-dioxane (50 °C)	**20**: 35%
**10**	**17, Pd(dba)**_**2**_ **, K**_**2**_ **CO**_**3**_ **, 1,4-dioxane**	**20: 85%**
11	**17**, Pd(dba)_2_, dppf, K_2_CO_3_, 1,4-dioxane	**20**: 22%

^a^The reactions were conducted at room temperature unless specified. All of the reported yields are isolated yields. EOM = ethoxymethyl; dppf = 1,1′-bis(diphenylphosphino) ferrocene.

**Figure 5:**
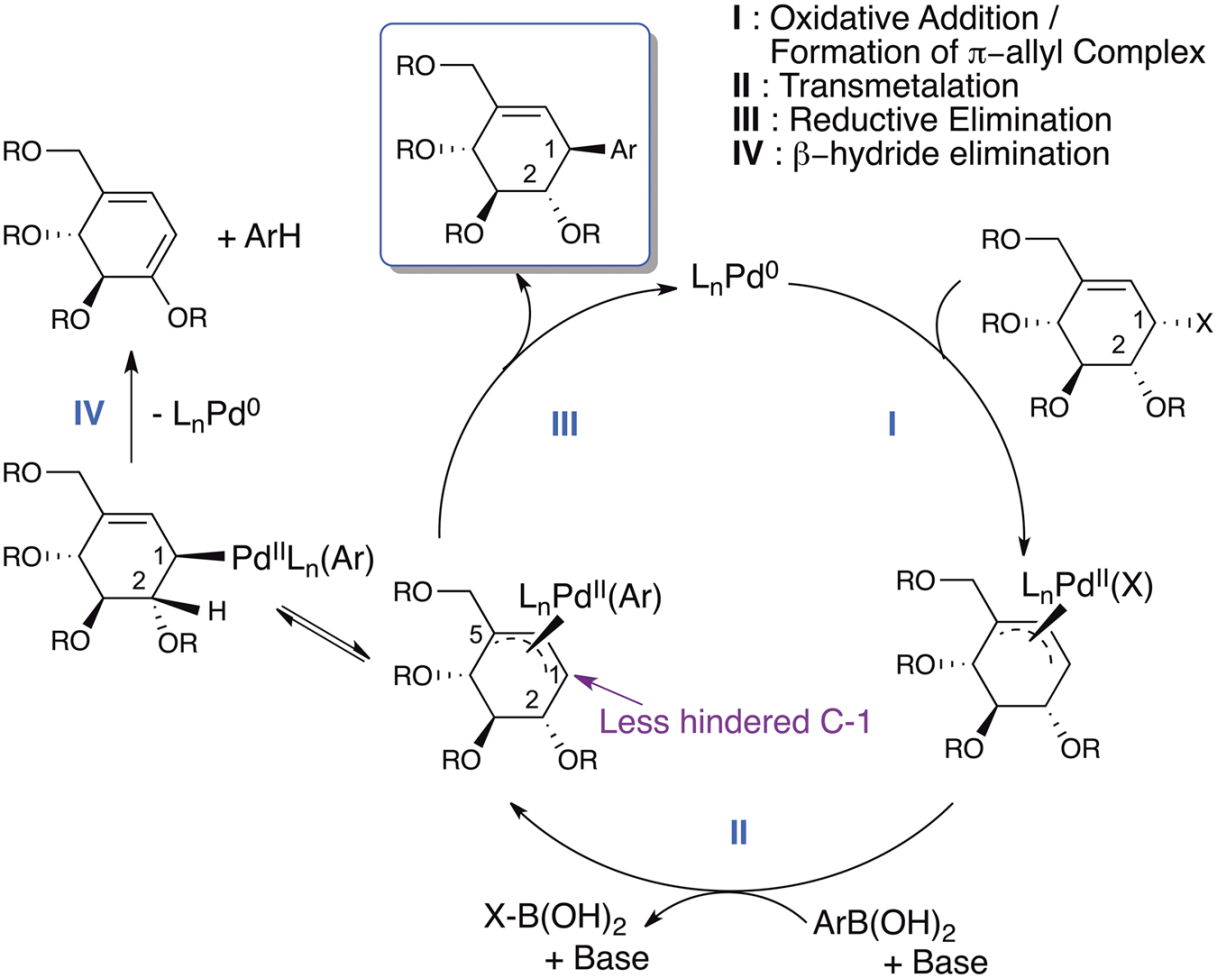
Proposed reaction mechanism of the allyl-aryl coupling reaction.

A plausible reaction mechanism is proposed to explain the excellent stereo- and regio-selectivities of the allyl-aryl coupling reaction (Fig. 5). A π-allyl Pd complex is first formed *via* a back-side attack from the less-hindered face (Fig. 5, pathway I). After the transmetalation step, the aryl group on the π-allyl Pd complex is delivered internally to the less-hindered C-1 position of the carbocycle, resulting a net-inversion of configuration and an exclusive regioselection (pathway III). On the other hand, syn-β-H elimination (pathway IV) of the η^1^-Pd complex is a viable degradation pathway, which is suppressed at a lower reaction temperture.

**Figure 6:**
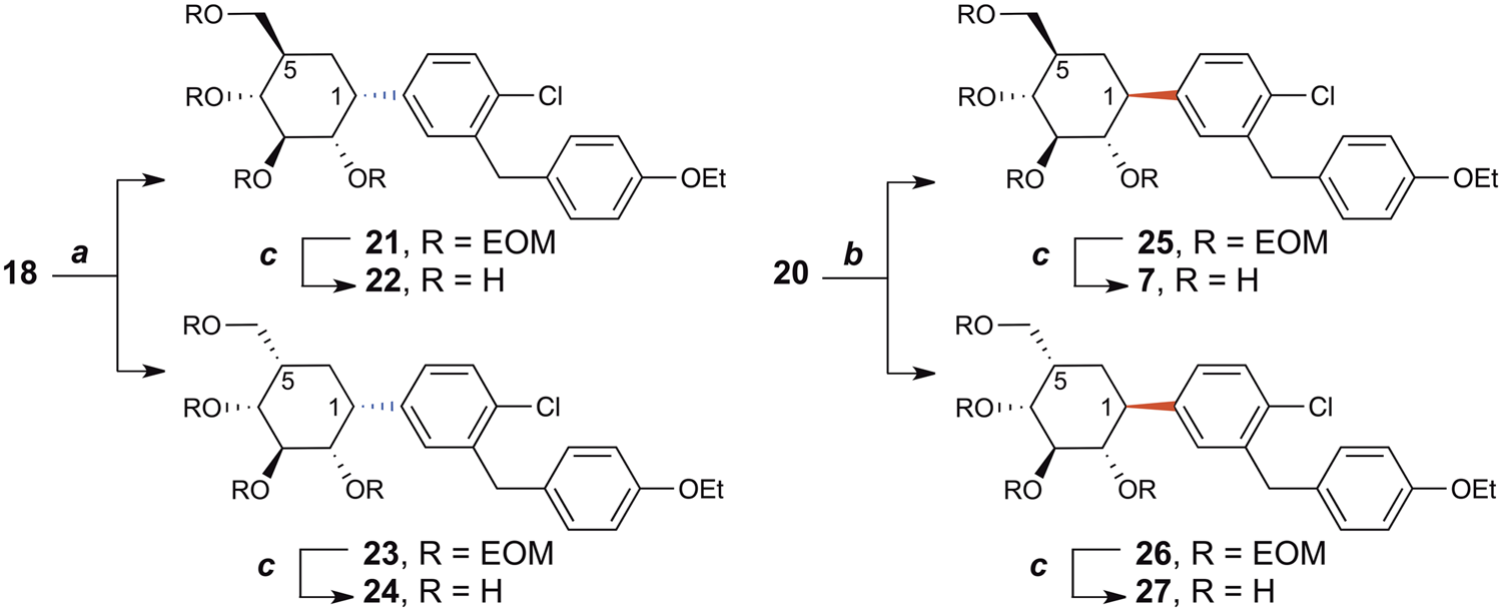
Stereodivergent syntheses of cyclohexane analogues of dapagliflozin. (**a**) *p*-TsNHNH_2_, NaOAc, THF_(aq)_, reflux; (**b**) *p*-TsNHNH_2_, NaOAc, THF_(aq)_, 70 °C; (**c**) HCl_(aq)_, EtOH, 50 °C, **22**: 51% from **18**; **24**: 7% from **18**; **7**: 65% from **20**; **27**: 25% from **20**.

Next, the arylated cyclohexenes **18** and **20** were subjected to alkene hydrogenation and global deprotection (Fig. 6). Our initial attempts of Pd-, Pt- or Ni-catalyzed hydrogenation led to complete dechlorination of the aglycone moiety. However, the diimide reduction of **18** and **20** was highly chemoselective: the alkene moiety was hydrogenated while the chloride substituent on the proximal benzene ring was preserved. This diimide reduction strategy also provided a stereodivergent entry to both C-1 and C-5 epimers of the cyclohexane analogues of dapagliflozin (Fig. 6). Thus, starting from the common precursor **15**, all four possible diastereoisomers (aryl pseudo-*C*-glycosides **22**, **24**, **7**, and **27**) were prepared for the subsequent SAR study. We also synthesized the aryl pseudo-*O*-glycosides **28**–**30** for a more comprehensive SAR study.(See Supporting Information)

### Structure-activity relationship (SAR) study

**Figure 7:**
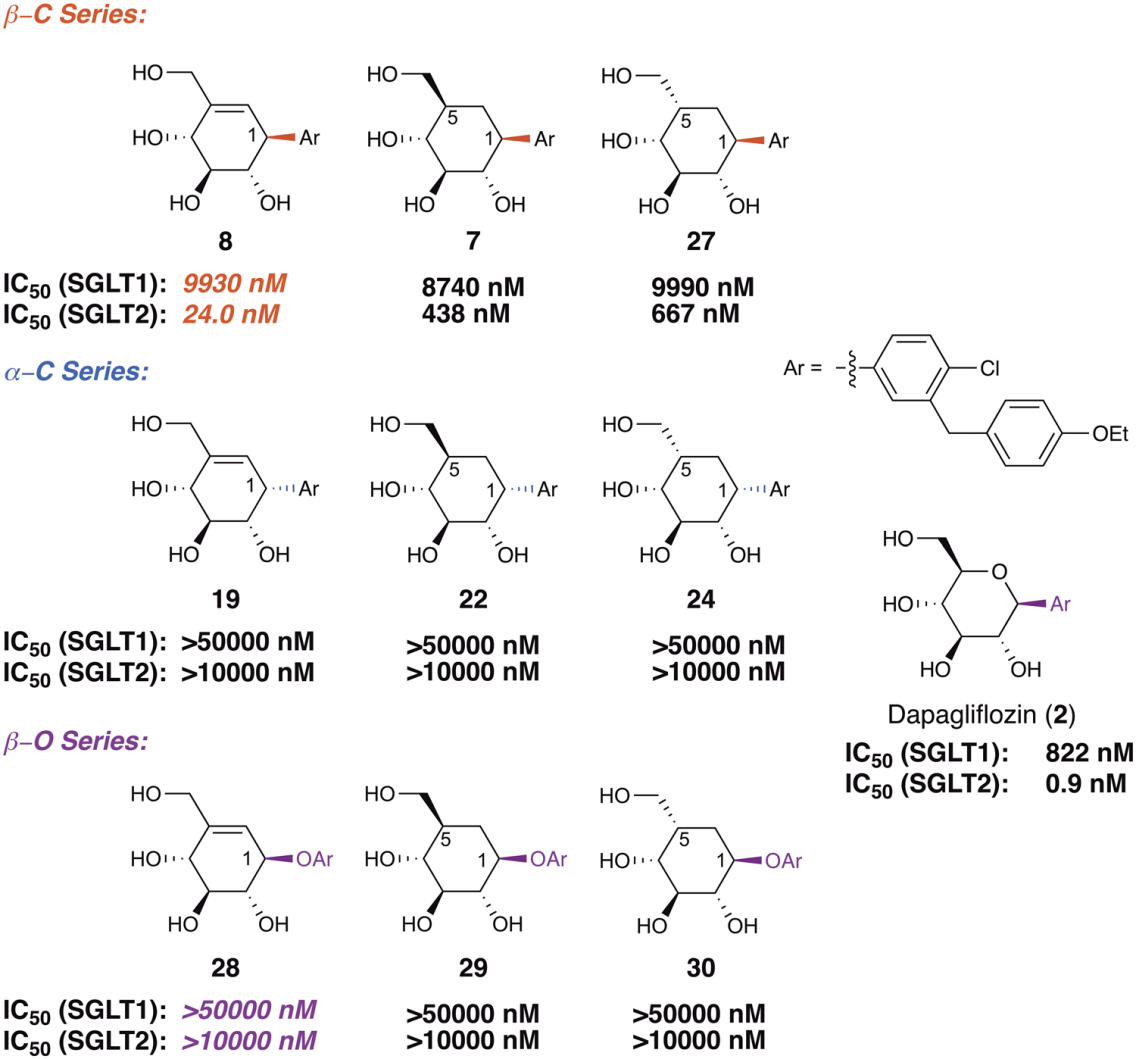
IC_50_ values for the novel analogues of dapagliflozin towards SGLT1 and SGLT2.

A cellular ^14^C-α-methyl-D-glucopyranoside (^14^C-AMG) uptake assay was used to evaluate the SGLT2/SGLT1 inhibitory activities of our carbasugar analogues of dapagliflozin (Fig. 7). In general, the β-C series were more active than the α-C series, hinting that the β-configuration at C-1 is critical for the inhibitory activity. However, within the β-C series, the cyclohexane analogue **7** showed only a moderate SGLT2 inhibitory activity and only 20-fold SGLT2/SGLT1 selectively; whereas the cyclohexene analogue **8** showed a nanomolar SGLT2 inhibitory activity and more than 400 fold SGLT2/SGLT1 selectively. The excellent potency and selectivity of **8** towards SGLT2, together with its enhanced stability (as a result of its dual C-C linkage), suggested that it is a highly promising lead compound for further optimization as a clinically useful SGLT2 inhibitor.

Another piece of unexpected SAR information came from the activity profiles of aryl pseudo-*O*-glycosides **28**, **29**, and **30** (β-O series), in which an exocyclic oxygen atom connects the carbasugar and aglycone moiety. We found that this subtle change in spatial connectivity is detrimental to the activity of carbocyclic analogues of dapagliflozin, as suggested by the dramatic loss of inhibitory activity in the β-O series.

### Computational modeling

**Figure 8:**
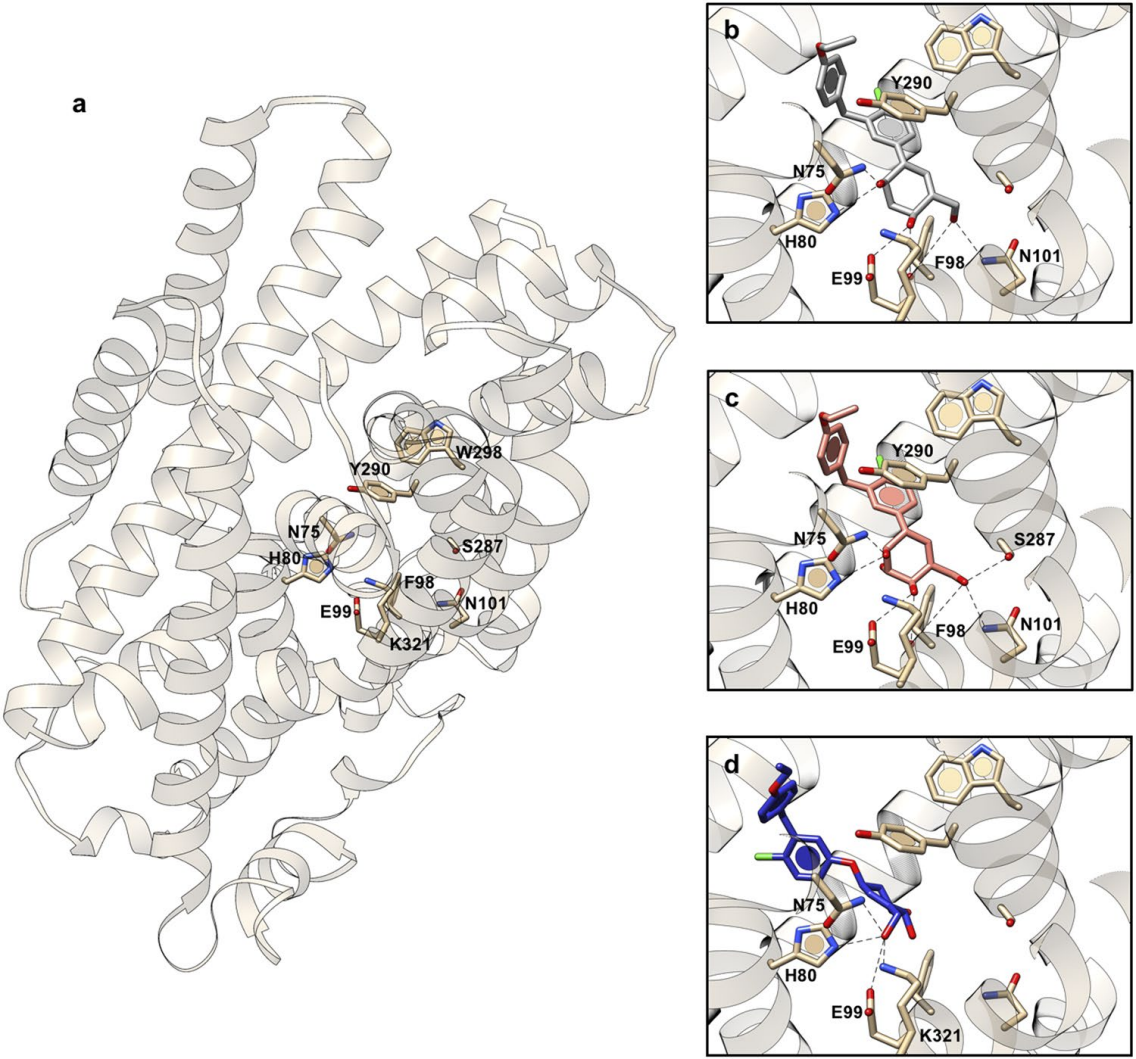
*In silico* docking analysis of hSGLT2-ligand interactions. (**a**) Ribbon representation of our hSGLT2 homology model. Molecular docking of (**b**) compound **7**, (**c**) compound **8** and (**d**) compound **28** into hSGLT2. Hydrogen bonds are shown as dotted lines, and key interacting residues are labelled.

The superior performance of cyclohexene **8**, together with the unexpected activity profile of the other carbasugars, prompted us to investigate the binding mode of carbasugar SGLT2 inhibitors by computational modeling. We performed *in silico* docking analysis by fitting different ligands into a homology model of SGLT2 (Fig. 8). As shown in our homology model, the ligand binding pocket of SGLT2 is shaped by the hydrophobic residues Phe98, Tyr290, and Trp298, together with hydrogen bond donors/acceptors such as His80, Glu99, and Asn101^36, 37^. (Fig. 8a) We found that the diarylmethane moiety in both **7** & **8** stacked perfectly with the aromatic ring of Tyr290 (Fig. 8b and c), resulting in strong π-π stabilizations. There are also extensive hydrogen bonding interactions between the carbasugar hydroxyl groups and His80, Glu99, and Asn101. Although the hydrogen bond network of **7** and **8** closely resembles each other, the distorted chair conformation of **8** allowed an additional hydrogen bonding interaction between the primary hydroxyl group and Ser287, thus leading to an enhanced binding affinity of **8**. (Fig. 8c) Our computational modeling also revealed that pseudo-*O*-glycoside **28** is a poor ligand of SGLT2 (Fig. 8d). The exocyclic oxygen atom in **28** effectively disrupts the π-π interactions and also withholds the carbasugar core from forming an extensive hydrogen bond network, resulting in the observed lack of SGLT2 inhibitory activity of **28**.

With the assistance of computational modeling, this SAR study has provided important insights into the ligand space of carbasugar SGLT2 inhibitors, thereby guiding the future exploration of these inhibitors as novel anti-diabetic agents. In addition, recent studies have suggested additional beneficial effects of SGLT2 inhibition, such as reducing body weight, blood pressure, cardiovascular and renal events^12^, which helped to uncover the hidden therapeutic potentials of our novel carbasugar SGLT2 inhibitors.

## Conclusions

In conclusion, we have developed a concise and stereodivergent synthetic route towards some novel carbasugar analogues of dapagliflozin. The key step involves a mild, regioselective, and stereospecific palladium-catalyzed allyl-aryl coupling reaction which has been underexploited in the synthetic community. A chemoselective diimide reduction was also critical to our synthetic route. Notably, this synthetic study revealed unexpected structure-activity relationship (SAR) of carbasugar SGLT2 inhibitors, and also enabled the discovery of the highly potent and selective inhibitor, cyclohexene **8**. Our study will therefore guide the future design and syntheses of carbasugar SGLT2 inhibitors, thereby unlocking their potential as novel diabetes therapeutics and beyond.

## Methods

### Preparation of compound 8

To a stirred solution of cyclohexene **20** (66.6 mg, 0.105 mmol) in EtOH (1.5 mL) was added 1 M HCl (aq) (1.5 mL) at rt. The reaction mixture was stirred at 50 °C for 3 h. Concentration of the reaction mixture under reduced pressure followed by flash chromatography (CHCl_3_:MeOH, 9:1) yield tetraol **8** (36.9 mg, 87%) as a white solid. For detailed characterization of **8**, please refer to supporting information.

### Preparation of compound 20

To a mixture of allylic carbonate **17** (71.7 mg, 0.154 mmol) and boronic acid **10** (141.1 mg, 0.486 mmol) in degassed 1,4-dioxane (0.19 mL) was added Pd(dba)_2_ (5.5 mg, 0.0096 mmol) and K_2_CO_3_ (68.1 mg, 0.493 mmol) sequentially. The reaction mixture was degassed for 3 times and stirred for 72 h at rt under nitrogen. Concentration of the reaction mixture under reduced pressured followed by flash chromatography (hexane: EtOAc, 7:2) yielded cyclohexene **20** (82.8 mg, 85%) as a colourless oil. For detailed characterization of **20**, please refer to supporting information.

### Procedure for ^14^C-α-methyl-D-glucopyranoside (^14^C-AMG) uptake assay

SGLT1- or SGLT2- expressing cells (S1 or S2) were developed by transfecting, respectively, SLC5A1 (NM_000343) or SLC5A2 (NM_003041) human cDNA clone (Origene Technologies Inc., Maryland, USA) into COS-7 cells (monkey fibroblast-like kidney cells, CRL-1651, ATCC, Manassas, USA). The cells were cultured in DMEM supplemented with 10% FBS, 1% PS and G418 (1 mg/ml), and kept in 37 °C humidified incubator supplied with 5% CO_2_. For the uptake assay, the cells (2 × 10^5^ cells/well) were seeded into 24-well plate and incubated overnight. Then, each well was added with the testing samples at different concentrations and 100 µCi/ml ^14^C-AMG. The cells were incubated in sodium buffer (140 mM NaCl, 2 mM KCl, 1 mM MgCl_2_, 1 mM CaCl_2_, and 10 mM Hepes/Tris, pH 7.5) at 37 °C for 2 hours. After incubation, the plates were washed three times with cold choline stop buffer (140 mM choline chloride, 2 mM KCl, 1 mM MgCl_2_, 1 mM CaCl_2_, and 10 mM Hepes/Tris, pH 7.5) containing 100 µM phlorizin dihydrate. After removing all buffer, the cells were then solubilized with 150 µL of NaOH (0.5 M) and followed by 150 µL of HCl (0.5 M). Then, 200 µL solution was taken out for the measurement of radioactivity and 30 µL solution was used to measure the protein content by BCA protein assay, which was used to normalize the cell number in each well. The amount of glucose uptake was expressed as cpm/mg protein.

### Homology Modeling of Human SGLT2

A homology model was generated from the structural template of sodium-galactose symport from *Vibrio parahaemolyticus* (PDB ID: 3DH4) (Faham *et al*., 2008). The homology modeling was performed with MODELLER (Sali and Blundell, 1993) in UCSF Chimera (Yang *et al*., 2012). UCSF Chimera alpha version 1.12 was used for the modeling. For validation of this homology model, please refer to supporting information.

### *In silico* Docking Analyses

The receptor macromolecule and ligands were prepared by using the Dock Prep tool in UCSF Chimera. During preparation, solvent and non-complexed ions were deleted, truncated sidechains were repaired, hydrogen atoms were added, partial charges were assigned, files were saved as Mol2 format. Docking was subsequently performed in UCSF Chimera (alpha version 1.12) using the AutoDock Vina tool version 1.1.2. The ViewDock tool in UCSF Chimera was used to analyze the receptor-ligand docking results. For details of the docking results, please refer to supporting information.
